# Assessment of Antimicrobial Potential of *Plagiochasma rupestre* Coupled with Healing Clay Bentonite and AGNPS

**DOI:** 10.1155/2022/4264466

**Published:** 2022-07-16

**Authors:** Muhammad Musa Khan, Qaisar Abbas Bhatti, Muhammad Akhlaq, Muhammad Ishaq, Daoud Ali, Aamir Jalil, Junaid Asghar, Saud Alarifi, Abdelhamid Elaissari

**Affiliations:** ^1^Depatment of Chemistry, Faculty of Natural Sciences, Mohi-Ud-Din Islamic University Nerian Sharif, Azad Jammu & Kashmir 12010, Pakistan; ^2^Faculty of Pharmacy, Gomal University, Dera Ismail Khan, Khyber Pakhtoon Khwa, Pakistan; ^3^Department of Zoology, College of Science, King Saud University, PO Box 2455, Riyadh 11451, Saudi Arabia; ^4^Department of Pharmaceutics, Faculty of Pharmacy, Bahauddin Zakariya University, 6800 Multan, Pakistan; ^5^Univ Lyon, University Claude Bernard Lyon-1, CNRS, Lyon, France

## Abstract

The impact of individual component, i.e., plant extract (*Plagiochasma rupestre*), biosynthesized silver nanoparticles (AgNPs), and healing clay (bentonite) as antimicrobial agent is reported but their combined effect as a ternary system is a new approach. This study is aimed at investigating the impact of the proposed ternary system against selected human pathogens. AgNPs were synthesized by using *Plagiochasma rupestre* extract (aqueous) as reducing agent and neutral polymer (PVP) as stabilizer. The morphology, size, and structural properties of synthesized AgNPs were determined with XRD and SEM analysis which showed spherical monomodal particles with an average particle size of 25.5 nm. The antibacterial and antifungal activities of the individual and nanoternary system were investigated. The phytochemical screening of plant extract showed the presence of alkaloids, flavonoids, phenol, and glycosides in methanol extract as compare to aqueous and acetone extract. The antimicrobial activities of crude extracts of *Plagiochasma rupestre* with AgNPs and bentonite clay were studied as an appropriate candidate for treatment of microbial infections, especially bacterial and fungal diseases. The antioxidant activity of *Plagiochasma rupestre* aqueous extract and nanoparticles was assessed by (DPPH) free radical, and absorbance was checked at 517 nm. Crude extract has inhibitory effect towards bacteria and fungi, and bentonite clay also showed some degree of antimicrobial resistance. Strategy can be efficiently applied for future engineering and medical. The nanoternary systems showed 3 and 3.5 times higher antibacterial and antifungal activity, respectively, in comparison to *Plagiochasma rupestre* and bentonite clay, individually.

## 1. Introduction

Development in the field of medicine has been remarkably progressed with the introduction of nanotechnology in the field of drug delivery, medical imaging, disease diagnosis, cancer treatment, and gene therapy and also to aid in visual imaging [1–3]. This cutting-edge technology is rapidly using in the development of healthcare product, and we are already experiencing its potential benefits on human health benefits, especially in the field of drug delivery nanoparticles which are used as tools to deliver different therapeutics including proteins, antibodies, and nucleic acids [4]. Most, importantly the emergence of antimicrobial resistance can be tackled by using these gold and AgNPs [5]. In the physics, the nanoelectronics, nanomechanics, nanophotonics, and nanoionics [6–9] were as follows; in the chemistry and metallurgy, the nanotopography, nanolithography, self-assembled monolayers, nanowire compositions, nanoenzyme nanodiamonds, and nanoclusters [1, 10–12] were as follows, and in the medical science, the compatibility of coexistence with living tissues, productivity, eccentric drug, and drug distribution [13] due to high surface volume ratio of nanoparticles is as follows.

In nanomaterials, nanometal is an important topic in the field of research and modern material science because of its unique properties in different fields of life: electronics, catalyst, dental material synthesis, and drug [14]. Due to this reason, the researchers showed great interest in the synthesis and application of metallic nanoparticles. Legislature arranges programs to attract people for this field.

Silver metal is the metal of choice among the noble metals in the field of biological system, living organisms, and pharmaceutical industry. AgNPs are more interesting in colloidal perpetrations because of their characteristic properties, like chemical conductivity, stability, catalytic, and antibacterial activities [15–17]. Silver as nanoparticles has been used in medicine for dental capping, wound treatment, coating on material objects, cleaning of water, and in sunscreen lotions ([18–20]. Silver has long been known as a sterile and antibiotic since having an inhibitory effect on many microorganisms [18, 20].

Among the different synthetic methods for the preparation of AgNPs such as sol-gel method, chemical precipitation, chemical vapor deposition, green synthesis, and hydrothermal method , and green synthesis of nanoparticles is more preferable because of its nontoxicity to environment, biodegradable approach, and eco-friendly nature [21, 22].

Keeping in view the advantages of green synthesis of AgNPs, the usage of different plant parts for the synthesis of AgNPs is a common practice. Although the bryophytes in this connection remained a neglected group of plants, yet this group has a very high antifungal and anitbacterial potential, because biological compound like phenyl quinene, aromatic and phenol substance, oligosaccharides, polysaccharides, sugar alcohols, and amino acids in bryophytes provides protection against these organism [23–26]). Among bryophytes, *Plagiochasma rupestre* is liverwort belong to family Aytoniaceae that is an appropriate candidate exhibiting interesting biological activates including antibacterial, antifungal, cytotoxic, insect repellent, insecticides, and muscle relaxing as well as posing some enzyme inhibitory activities [26–31]. For therapeutic purposes, the *Plagiochasma rupestre* thallus was resistant against microorganisms [25, 26, 30, 32]. It was suggested that the aqueous extract of plant contains antioxidant properties and antimicrobial activity. *Plagiochasma rupestre* is found at lower elevation in arid areas. It grows on exposed rocks and soil surface. This species is well known from all the bio-geographical regions of the world [26, 30, 33].

Not only the phytoremediation but the use of minerals as medicine is also very old. Among the minerals, clay minerals like bentonite and smectite kaolinite are used in pharmaceutical formulations in the form of powder, suspension emulsions, or gel due to their high specific area, chemical inertness, rheological properties, high sorptive capacity, and low or null toxicity for the patient. The applications of clay are not limited to therapies like topical applications (dermatological protectors, cosmetics). Pharmaceutical preparations, using minerals as carrier releasers of active ingredients, are very important for environmental technology for the protection of our environment against dangerous substances [24, 34–40].

Among phyllosilicates, clay bentonite is a natural nonsymmetrical porous clay with a particular composition of silicone magnesium, and aluminum is beneficial to human health such as in pharmaceutical preparation, medicinal therapy, and beauty therapy due to cytoprotective action [34, 35].

Keeping in view the individual applications of all above three components, a novel ternary system against the human pathogens is proposed. The aim of the present study was to investigate and compare the pharmacological properties like antimicrobial and antioxidant activities of individual components and their novel ternary system comprising of AgNPs synthesized from aqueous extracts of *Plagiochasma rupestre* and bentonite clay against selected pathogens.

## 2. Material and Methods

### 2.1. Collection of Plant Sample

**Figure 1:**
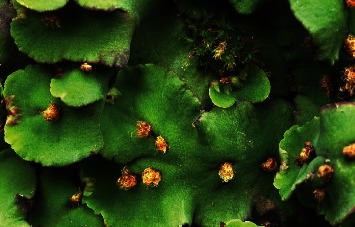
*Plagiochasma rupestre* [40].

The plant *Plagiochasma rupestre* was collected in the hilly area of Nerian Sharif (Tarar Khel, longitude 33.9259°N, latitude 73.7810°E) district Sudhnoti, Azad Jammu and Kashmir, Pakistan, in the month of September 2020 (Figure 1) and identified by experts from the Department of Botany at Mohi-Ud-Din Islamic University, as *Plagiochasma rupestre*, and the specimen was for later reference kept in the herbarium of the department.

### 2.2. Bentonite

Bentonite USP-type (Sigma) having very fine particles was used as model substrate. Some of the characteristics of bentonite are presented in Table 1. These properties were also verified by employing a mercury porosity meter (Autosorb-1 der Firma Quanta chrome, USA), pH, laser diffraction measurements, and scanning electron microscopy.

### 2.3. Preparation of Plant Extracts

#### 2.3.1. Aqueous Extract

Aqueous extract of shade dried *Plagiochasma rupestre* was prepared by simple maceration. Plant material (50 g) was ground and soaked in distilled water (200 ml). The mixture was boiled for 30 min until the color of the solution changes to light yellow. The extract was cooled and filtered through Wattman filter paper No. 1. The filtrate was centrifuged for 10 minutes at 4000 rpm for the removal of suspended material. A crude extract was then obtained which is stored at 4°C until use.

#### 2.3.2. Methanolic/Acetonic Extract

Methanolic extract of shade dried *Plagiochasma rupestre* was prepared by simple maceration. Plant material (50 g) was ground and soaked in absolute methanol (200 ml) and acetone (200 ml) for fourteen days, and the extract was concentrated by the Rotavapor model (Rotavapor R-220 Pro) obtain as crude extract and stored at 4°C until use.

### 2.4. Bentonite Dispersion

Aqueous dispersion was prepared by dispersing 0.1 g of bentonite in to 100 ml of Millipore water (0.1% solid content), using a high intensity ultrasonic processor, UP-200 s operating at 24 kHz, up to 200 W (Dr. Hielscher GmbH), at 22°C. The dispersion was characterized by different standard experimental techniques [41].

### 2.5. Synthesis of AgNPs

Silver nitrate (Sigma-Aldrich) was taken as a precursor salt for the synthesis of the AgNPs. In order to achieve the optimum concentration of AgNPs, the plant extract was treated with different concentrations of AgNO_3_ solution varying from (0.001 M to 0.1 M) in flasks and incubated at room temperature in a dark place. The color change was observed after the intervals of 24 hours till 72 hours. The solution turned out to be dark brown suggesting the formation of AgNPs. In order to find the maximum synthesis, the mixture was observed till 72 hrs when no more change in absorbance was observed. The pH of the solution was maintained to neutral by using NaOH and HCl (Sigma-Aldrich, Merck). Then, colloidal dispersion then subjected to centrifugation (centrifuge model OSK12710 (Gawasieki Co., Japan)) for purification purpose by utilizing double distilled water. The solution was centrifuged (3-times) at 4,000 rpm for 15 minutes, and the supernatant was replaced by distilled water each time. The collected supernatant is dried and calcinated at 300°C for 3 hrs.

Synthesized AgNPs were then characterized by different spectroscopic techniques like UV-Vis spectroscopy, XRD analysis, scanning electron microscopy analysis (SEM), and Fourier transform infrared spectroscopy (FTIR).

### 2.6. Biological Activities

The following biological activities were carried out on Plagiochasma rupestre plant extracts, AgNPs, and bentonite clay, as well as the ternary system composed of these three components.

#### 2.6.1. Antibacterial Assay

The antibacterial assay was carried out using the disc diffusion technique, as reported by Bibi et al. [42]. Antibacterial activity was tested against two bacterial strains: gram-positive (*Bacillus subtitles* (MTCC619) and gram-negative *Salmonella typhimurium* (MTCC98). In brief a 100 *μ*l of 24-hour-old bacterial cultures in nutrient broth were dispersed onto Petri plates (7 cm) containing 25 ml solidified nutritional agar. With the use of sterilized forceps, sterile filter paper discs (diameter 6 mm) impregnated with 100 *μ*l of extract dilutions, AgNPs, and bentonite clay solution (1, 5, and 10 mg/ml DMSO) were put over each of the culture plates. The plates were then incubated for 24 hours at 37°C. The zones of inhibition surrounding the discs in each plate were measured to evaluate antibacterial activity. As a positive control, paper discs impregnated with 100 *μ*l of a 2 mg/ml were as follows. Ciprofloxacin solution was employed, while DMSO was used as a negative control. The test was carried out in triplicate, and the mean with standard deviation was computed.

#### 2.6.2. Antifungal Assay

Antifungal assay was performed as described by Jagessar et al. [43]. Antifungal activity was studied against two fungal strains, namely, *Aspergillus niger* (FFBP 0198) and *Rizopus oryzae* (FFBP 0291). All fungal strains were grown on 6.5% SDA (Sabouraud dextrose agar, pH 5.7) at 28°C and preserved at 4°C in refrigerator. Iitraconazole (200 *μ*g/ml) was used as standard drug while DMSO was used as negative control. Three concentrations of each of the extracts, i.e., 1, 5, and 10 mg/ml DMSO were tested. SDA plates containing 25 ml medium were inoculated with 100 *μ*l fungal spore suspensions which was prepared by harvesting fungal spore's suspension in 2% w/v tween 20 solutions in distilled, and turbidity was adjusted to 0.5 McFarland turbidity standards. The inoculator has been stored for further use at (4°C). Inoculator was diluted on solid potato Dextrose agar checks the absence of contamination and verifies the inoculums' validity. Inoculated plates were then incubated at 28°C for 24 h, and zones of inhibition were measured in millimeter (mm) around the each disc.

#### 2.6.3. Free Radical Scavenging Activity

The scavenging ability of the free radical DPPH was evaluated by the antioxidant assay of Plagiochasma rupestre extract and AgNPs. Aqueous extracts of Plagiochasma rupestre and AgNPs of extract prepared (25-400 mg/ml) ethanol were added, and DPPH solution was mixed and shaken. After that, the mixture was kept in dark for 30 minutes. Using the Cecil-Elect UV Spectrophotometer at 517 nm against blank (1 ml ethanol+0.5 ml DPPH solution), optical mixture density was calculated. Optical density was calculated for each sample. Both readings in triplicates were taken. Scavenging ability was determined by the following formula.

(1) $$\begin{array}{c} \%\text{scavenging effect}=\left(1-\text{As}/\text{Ac}\right)\times 100, \end{array}$$

where “Ac” is the absorbance of negative control, and “As” is the absorbance of test sample.

### 2.7. Phytochemical Screening

Phytochemical screening of aqueous, methanol, and acetone extracts of *Plagiochasma rupestre* was performed in order to recognize the constituents. Standard qualitative tests were performed.

### 2.8. Statistical Analysis

For data analysis, experiments were independently replicated at least three times and completely randomized design (CRD), and one-way ANOVA was used with three replicates.

## 3. Results and Discussions

### 3.1. Synthesis and Characterization of AgNPs

AgNPs were prepared according to the standard procedure mentioned in the literature. Characterization is carried out on synthesized nanoparticles through UV-Vis spectroscopy and FTIR. The effect of pH, time influence on pH, and the effect of plant extract concentration and silver nitrate on AgNP synthesis have also been studied.

### 3.2. UV-Visible Analysis for the Synthesis of AgNPs

**Figure 2:**
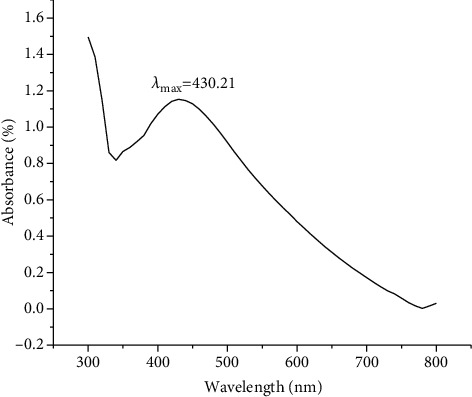
Uv-Vis spectra of *Plagiochasma rupestre* nanoparticles.

With the help of UV-Vis spectrometer with a resolution of 0.5 nm from 190 nm to 1100 nm, UV-visible spectroscopic research was carried out.  Figure 2 indicates the evolution of the reaction between metal ions, and the plant extract was monitored by UV-visible AgNP spectra in aqueous solution with different reaction times (0 to 72 hours) (R [44]). At 430.2 nm, the efficiency surface plasmon absorption band was fascinated, suggesting that silver ion reduction and stable AgNP formation occurred rapidly within an hour of reaction and completed after 72 hours.

#### 3.2.1. Optimum Condition/Concentration for the Synthesis of AgNPs

**Figure 3:**
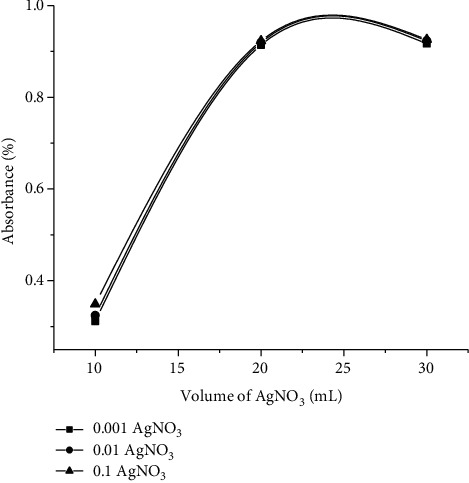
Effect of volume of plant extract on the synthesis of AgNPs.

Various ratios of plant extract (10-30 ml) were reacted with a constant volume of AgNO_3_ with a concentration range of (0.001 M to 0.1 M) in order to achieve the optimal conditions for the synthesis of AgNPs, and subsequent analysis was carried out at room temperature. The ratio of plant extract with the concentration of AgNO_3_ is shown in Figure 3, and UV-visible spectra of AgNPs in aqueous solution with different reaction times (0 to 72 hours) were monitored for the production of the reaction between metal ions and plant extract, as shown in (Figure 3).

At 430 nm, the efficiency surface plasmon absorption band was fascinated, suggesting that silver ion reduction and stable AgNP formation occurred rapidly within an hour of reaction and completed after 72 hours. The graph is shown that the maximum amount of AgNPs produced was observed for the AgNO_3_ (0.1 M) and 30 ml of plant extract. It is represented from the graph that the synthesis of AgNPs increases with the increase concentration of AgNO_3_. The maximum amount formed was observed for the highest concentration of salt (0.1 M). By increasing the concentration of AgNO_3,_ the amount of silver ions increases; so, more reduction occurs.

The maximum amount formed was observed for the highest concentration of salt (0.1 M). The graph also showed that the synthesis is also time and volume of plant extract-dependent. The maximum volume of plant extract observed is 30 ml and 72 hours. Afterward, no significant increase in absorption band was observed. The increase in % absorbance became constant after 72 hours, which reflects that the reduction of Ag ions increases with the passage of time and becomes constant after 72 hours [45].

#### 3.2.2. Impact of Quantity of Plant Extract on AgNPs Synthesis by Maintaining a Constant Concentration of AgNO_3_ (0.1-0.01-0.001)

By increasing the quantity of plant extract, keeping the concentration of AgNO_3_ (0.1-0.01-0.001) constant, the %absorbance increases, and maximum absorbance was observed for 0.1 M AgNO_3._

#### 3.2.3. Impact of pH on Synthesis of Silver Nano Particles

**Figure 4:**
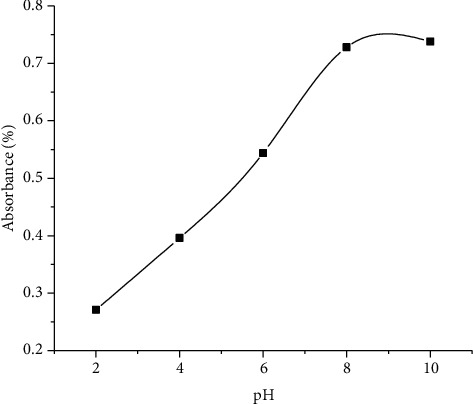
Impact of pH on AgNP synthesis.

Figure 4 indicates the effect of pH on the synthesis of AgNPs. It is observed that with increasing the pH, the % absorbance increases. As discussed earlier, increasing and shifting of the absorption band towards higher wavelength indicate not only the formation of AgNPs but also the reduction in particle size and vice versa. The absorption increased with the rise in pH, and the narrow peak at pH 10 showed the uniform distribution of particle size, while the peak becomes wider in the acidic pH range and the particle size increases. The absorption increases with an increase in pH and gives a narrow peak with uniform size distribution at pH 9. It is therefore presumed that the basic condition is preferential not only for the synthesis of AgNPs but also for particle size measurement [38].

A possible explanation for this outcome was that with subsequent crystallization into smaller particles, the reaction rate would be increased during high pH, which concerned the nucleation and growth processes of smaller Ag nuclei particles. The pH is inversely proportional to the size of AgNPs in general.

#### 3.2.4. Impact of Time-Dependent pH

**Figure 5:**
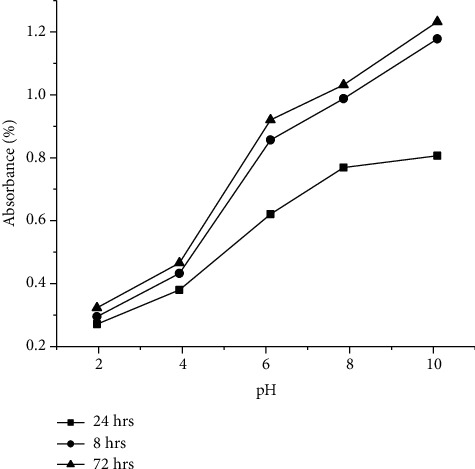
Impact of time-dependent pH.

Impact of time-dependent pH on the synthesis of AgNPs is representing in Figure 5. It is observed that not only with increasing the pH but also with increasing the time of contact between plant extract and AgNO_3_ solution, and the % absorbance increases. Maximum absorbance is observed after 72 hours in basic medium having pH 10. The results obtained are in good agreement with those reported by [46]. Hence, it is assumed that the basic condition and time of contact (72 hrs) are favored not only for synthesis of AgNPs but also for controlling the size of the particles.

### 3.3. Scanning Electron Microscopy

**Figure 6:**
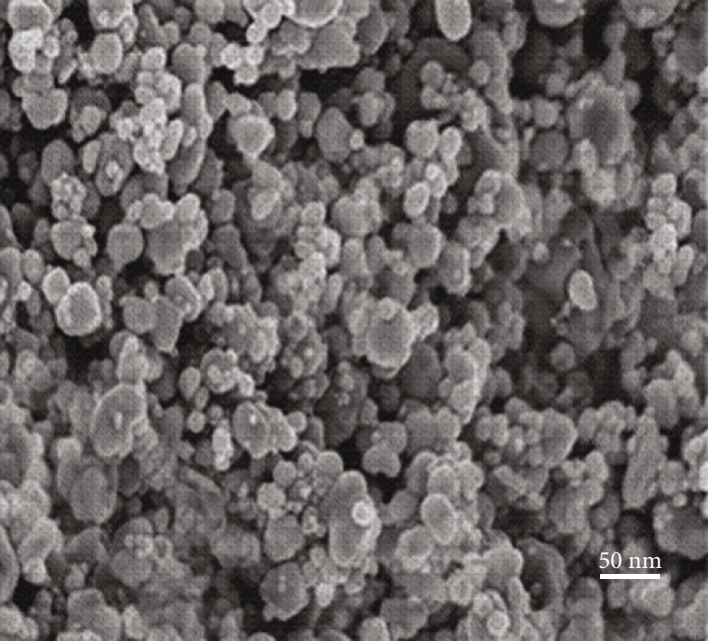
SEM picture of AgNPs.

Scanning electron microscopy is used to study the surface morphology of AgNPs at 100 nm that are synthesized. There were differences in their size in the SEM picture of AgNPs (*Plagiochasma rupestre*) (Figure 6). Particle SEM analysis shows that particles are in spherical shape.

### 3.4. Characterization of Bentonite

**Figure 7:**
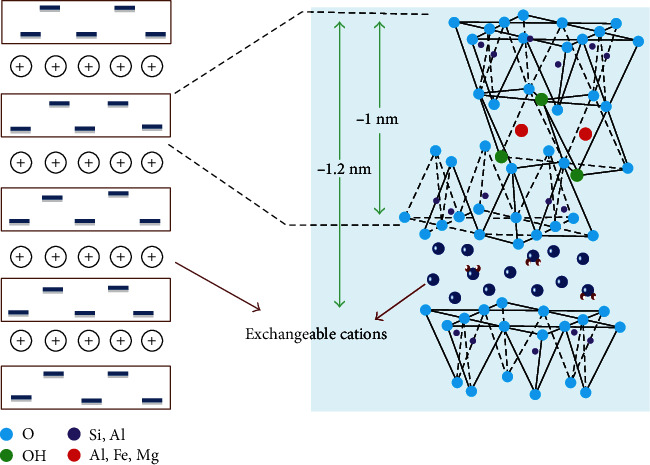
Schematic presentation of structure of bentonite clay [48].

Guven [47] suggested bentonite characteristics. It consists of two Si-oxygen tetrahedral sheets embedded in one-Al-oxygen octahedral sheet that is frequently used by mutual oxygen atoms linked to other two sheets; henceforth, the other name 2 : 1 layer structures are shown in Figure 7.

#### 3.4.1. Scanning Electron Microscope of Bentonite Clay

**Figure 8:**
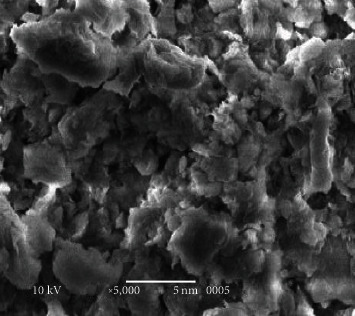
SEM picture of bentonite.

Scanning electron microscopy data for the bentonite sample could be potted as follows, occurring either as thin, small, leaf-like crystals forming a condensed aggregate,or in a more open surface of the honeycomb [49]. The bentonite SEM micrograph used for the evaluation shows its sheet structure as shown in Figure 8.

#### 3.4.2. Particle Size

The average particle size measured by (XRD) was 114.56 nm. From the peak values and calculations using Scherrer equation, it was found that the particle size distribution was narrow.

#### 3.4.3. XRD Analysis of Bentonite and AgNPs

Bentonite clay from Campina Grande, Paraiba, via XRD, is defined by [50, 51]. The patterns suggest the presence in the samples of diffraction peaks corresponding to planes (001) and (020), confirming the presence of montmorillonite. The data also indicate the presence of impurities in the measurement of bentonite clay, such as kaolinite (11.73 and 23.45o) and quartz (20.87 and 26.67o),

Similar XRD findings were obtained by (Silva et.al) when characterizing bentonite clay from the town of Pedra Lavada, Paraiba.

**Figure 9:**
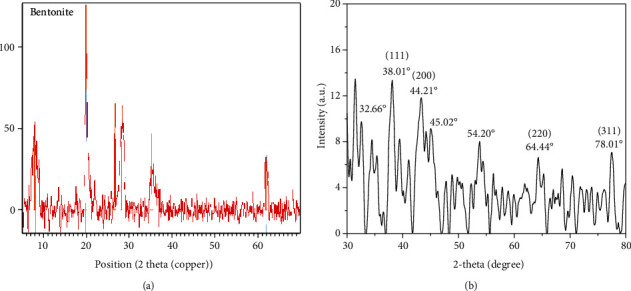
(a) XRD analysis of bentonite clay. (b) XRD analysis of AgNPs.

Bentonite is characterized by X-ray diffraction spectroscopy and, as shown in the above literature, similar results have been obtained (Figure 9(a)).

XRD analysis is performed to evaluate the crystalline nature and size of silver nanoparticles synthesized from aqueous extract of Plagiochasma rupestre. XRD patterns of the synthesized AGNPs are shown in Figure 4. The X-ray diffractogram peaks positioned at 38.01°, 44.21°, 64.4°, and 78.01° with d-spacing of 2.467, 5.7658, and 2.922 Å corresponded to the hkl plans of (111), (200), (220), and (311), respectively, indicated the synthesis of silver nanoparticles as shown in Figure 9(b) [52, 53].

#### 3.4.4. Point of Zero Charge and pH

**Figure 10:**
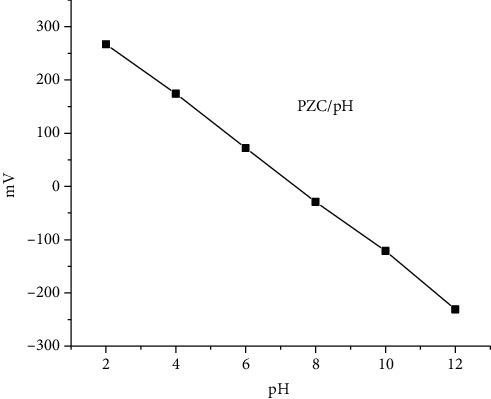
PZC in dependence on pH.

This potentiometric method was used as a pH function for the estimation of PZC. The PZC was estimated at pH 7.5 (Figure 10). Evidence of the adsorption and stability of dispersions has been given by the point of zero charge. The high charge density sample shows the highest stability of its dispersion.

#### 3.4.5. FT-IR Spectroscopic Measurements

**Figure 11:**
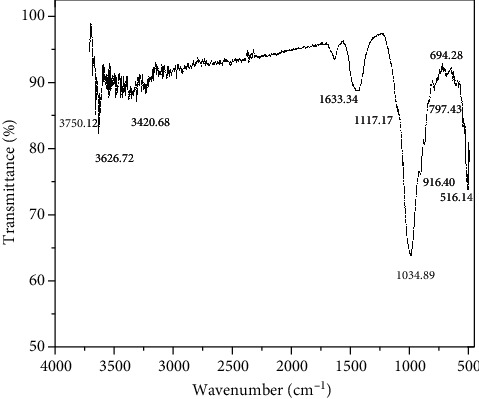
FTIR spectrum of bentonite.

FT-IR spectroscopy has been conducted to consider the structure, reactivity, and bonding of clay minerals. Evidence for the basic vibration mode of the constituent parts of bentonite was given by FTIR spectra in the 4000-500 cm^−1^ range. In the spectral region of 3750-3400 and 1633 cm^−1^, respectively, OH stretching and bending vibrations appear. Si-O-Si is indicated by strong absorption band at 1034 cm^−1^, while Si-O and Al-O bending modes dominate in the 516-916 cm^−1^ region [54]. The absorption band at 1117 cm^−1^ is an indication of calcite and absence of carbonates [55]. As mentioned above, Figure 11 shows similar results.

### 3.5. Biological Activities

#### 3.5.1. Antibacterial Activity

In ternary system AgNPs (*Plagiochasma rupestre* extract and bentonite clay), antibiotic potential of AgNPs against *Salmonella typhimurium* and *Bacillus substiles* is more (13-13 mm) as compared to crude extract (5-4 mm) and bentonite clay (1-3 mm), respectively, when the components are studied individually. Ternary system of extract, nanoparticles, and bentonite clay is more efficient in antibacterial potential (15-14 mm) than the binary system of bentonite clay and nanoparticles (14-14 mm), and clay and plant extract (4-4 mm) [56].

The antibacterial potential of *Plagiochasma rupestre* extract (aqueous, methanol, acetone), bentonite clay, and AgNPs observed individually and collectively. The results revealed that methanol extract of *Plagiochasma rupestre* has greater antibacterial potential (5-4 mm) then aqueous (2.5-2 mm) and acetone (1.5-1 mm) against *Salmonella typhimurium* and *Bacillus substiles* bacterial stains, respectively, as shown in the table [57].

**Figure 12:**
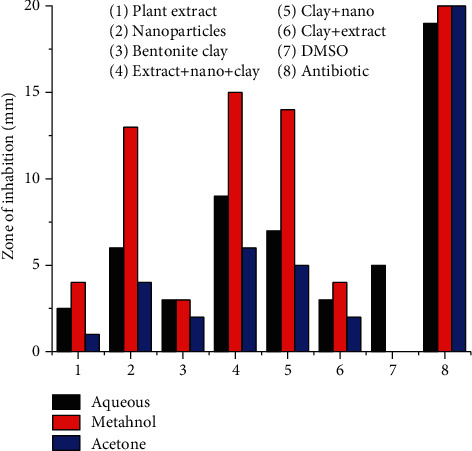
Antibacterial potential of Plagiochasma rupestre extracts, AgNPs, and bentonite clay against Bacillus.

**Figure 13:**
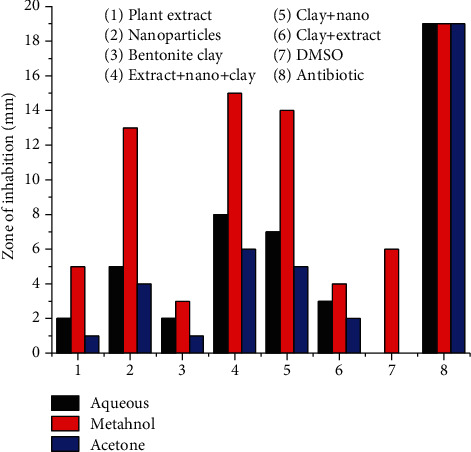
Antibacterial potential of Plagiochasma rupestre extracts, AgNPs, and bentonite clay against Salmonella.

The pictorial presentation of antibacterial effect of the ternary system, graphically the trend, is presented in Figures 12 and 13.

#### 3.5.2. Antifungal Activity

In ternary system AgNPs (*Plagiochasma rupestre* extract and bentonite clay), antifungal potential of AgNPs against *R. oryzae and A. niger* is more (20-20 mm) as compared to crude extract (10-12 mm) and bentonite clay (2 mm), respectively, when the components are studied individually. Ternary system of extract, nanoparticles, and bentonite clay is more efficient in antibacterial potential (22-22 mm) than the binary system of bentonite clay and nanoparticles (21-21 mm), clay, and plant extract (13-12 mm) [56].

The antibacterial potential of *Plagiochasma rupestre* extract (aqueous, methanol, acetone), bentonite clay, and AgNPs observed individually and collectively. The results revealed that methanol extract of *Plagiochasma rupestre* have greater antibacterial potential (12-10 mm) than aqueous (1-2 mm) and acetone (0-3 mm) against *R. oryzae and A. niger* fungal stains, respectively, as shown in the table [58].

**Figure 14:**
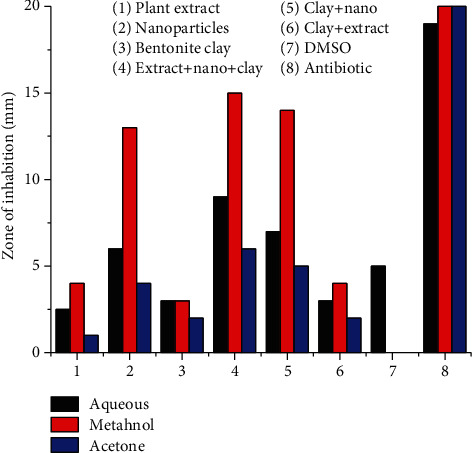
Antifungal potential of Plagiochasma rupestre AgNPs and bentonite clay against *Aspergillus niger.*

**Figure 15:**
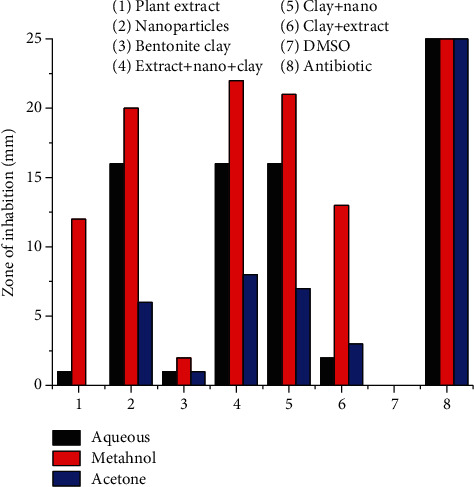
Antifungal Potential of Plagiochasma rupestre AgNPs and bentonite clay against *Rizopus oryzae.*

The pictorial presentation of antibacterial effect of the ternary system is graphically presented in Figures 14 and 15.

#### 3.5.3. Antioxidant Assay (Free Radical Scavenging Assay)

**Figure 16:**
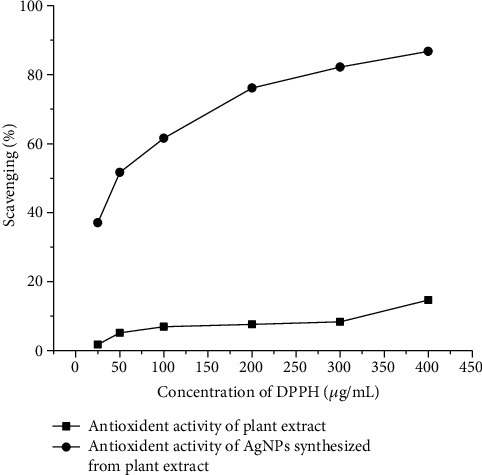
The antioxidant activity of *Plagiochasma rupestre extract* and silver nanoparticles synthesized from *Plagiochasma rupestre extract.*

The free radical scavenging activity of plant extract and crude extract AgNPs was measured spectrophotometrically by measuring the disappearance of DPPH at 517 nm as shown in Figure 16. The antioxidant testing results showed that AgNPs from plant extract had much higher antioxidant activity than control plant extract at all doses tested. Among all materials examined, AgNPs had the most antioxidant activity with an IC60 value of 86.754 g/ml, whereas Plagiochasma rupestre extract had the highest antioxidant activity with an IC60 value of 53.53 g/ml.

## 4. Discussion

Nanomaterials have shown to be an effective method for medication delivery in current research [31]. The antimicrobial properties of Ag ions and Ag-based compounds are well known, and many researchers are interested in employing other inorganic nanoparticles as antibacterial agents [59]. In recent years, the usage of medicinal plants has been revolutionized for the synthesis of AgNPs, which serves as an alternative to antibiotics and is an ecofriendly, nonhazardous, and cost-effective strategy [60]. Geophagy has been identified as an adaptive activity in humans and animals [61], and clays have long been regarded as therapeutic materials. Bentonite is an aluminum phyllosilicate clay that is highly absorbent. It has been utilized and consumed since ancient times because humans believe in its curative effects. When combined with water, it forms a paste that has been used both externally and internally. Because this clay is plentiful, inexpensive, and natural, it has been widely employed for a variety of medicinal reasons. There is a substantial body of data demonstrating its safety after chronic oral ingestion [62, 63].

In the current work, an aqueous extract of Plagiochasma rupestre was employed to synthesize AgNPs and compare them to bentonite clay against several biological activities (antimicrobial, antioxidant). When an aqueous extract was treated with silver nitrate solution, the solution's color changed from yellow to dark brown, confirming the production of AgNPs ([64]. Characterization of green synthesized AgNPs was done by using UV-Vis spectroscopy, FTIR, XRD, and SEM analysis as reported by [65]. Uv-Vis spectroscopy was done at the wavelength of 300-700 nm, that showed the characteristics peak obtained at 430.2 ((Figure 2) similar to the results of *Eucalyptus hybrid* [66]. FT-IR spectroscopy has been conducted to consider the structure, reactivity, and bonding of clay minerals. Evidence for the basic vibration mode of the constituent parts of bentonite was given by FTIR spectra in the 4000-500 cm^−1^ range. In the spectral region of 3750-3400 and 1633 cm^−1^, respectively, OH stretching and bending vibrations appear. Si-O-Si is indicated by strong absorption band at 1034 cm^−1^, while Si-O and Al-O bending modes dominate in the 516-916 cm^−1^ region [29]. The absorption band at 1117 cm^−1^ is an indication of calcite and absence of carbonates [55]. As mentioned above, Figure 12 shows similar results.

Presence of AgNPs in plant extract can be detected by determining the diffractions peaks by using XRD that is used to check the crystallite of nanoparticles. The nanoparticles of silver by using Plagiochasma rupestre showed peak (Figure 10). XRD findings of AgNPS are in agreement with the reported literature [24, 36]. The observed values are the typical of AgNPs, and the nanoparticles synthesized were of average size of 24 nm. [37]). Some additional diffraction lines positioned at 54°, 45°, and 32°, respectively, indicted the presence of other crystalline compounds in plant extract [24, 36]. However, a lot of aggregation is also observed which is due to settling of particles. Scanning electron microscopy is used to study the surface morphology of AgNPs at 100 nm that are synthesized. There were differences in their size in the SEM picture of AgNPs (Plagiochasma rupestre) (Figure 6). Particle SEM analysis shows that particles are in spherical shape.

In the current work, a crude aqueous extract of Plagiochasma rupestre was produced, and AgNPs were synthesized from this extract and tested for biological activity (antimicrobial, antioxidant) in comparison to bentonite clay. Green produced AgNPs from Plagiochasma rupestre, and bentonite clay had substantial antibacterial activity against all bacterial strains evaluated in an antimicrobial experiment, but crude extracts showed no significant antibacterial activity. AgNPs had the highest antibacterial activity when compared to plant extract and bentonite clay. Sereemaspun et al. and Shirley et al. [67, 68] found similar findings in Argimone maxicana, Allium cepa, Artocarpus heterophyllous, and Boswellia ovalifoliolata. According to reports, all bacteria employ an enzyme (protein) as a type of chemical lung to metabolize oxygen. Silver ions damage the enzyme and prevent it from absorbing oxygen. This successfully suffocates any bacterium, killing it in 6 minutes while leaving surrounding tissue or material unharmed [69]. At all doses examined, all plant extracts containing AgNPs and bentonite clay shown considerable antifungal activity. At all doses, the highest activity was seen against Aspergillus niger. When compared to binary and their individual components, the ternary system had the highest antibacterial activity. The findings showed that AgNPs may have antifungal effect by changing the structure of the cell membrane and impeding normal budding owing to membrane integrity damage. The findings indicate that AgNPs and bentonite clay might be a useful source of antibacterial compounds that need further research for therapeutic applications [70].

Natural clay minerals include trace metals such as Al, Ag, Cu, Mn, Zn, and Fe that may interact with microbial cell membranes and affect bacteria. The antioxidant capacity of the samples was assessed using the DPPH free radical scavenging test. Using various organic solvent systems such as ethanol, aqueous acetone, methanol, aqueous alcohol, and benzene, this test has been used to investigate the antioxidant capabilities of wheat, vegetables, herbs, edible seed oils, conjugated linoleic acids, and flours [71–74]. It is a nonenzymatic approach that is currently utilized to offer basic information about a compound's potential to scavenge free radicals. The reduction of DPPH by an antioxidant causes a drop in absorbance at 517 nm [75]. Significant DPPH scavenging action was seen in plant extract AgNPs as compared to control plant extracts at all doses tested, which is consistent with the findings of [76]. AgNPs had the greatest antioxidant activity of any material tested, followed by crude extract (See Figure 12).

## 5. Conclusion

When compared to its individual components, the ternary system demonstrated a considerable increase in antibacterial, antifungal, and antioxidant activity. This might explain why AgNPs and bentonite clay have a considerable favorable influence on Plagiochasma rupestre's various biological activities and phytochemistry. Furthermore, the current work implies that AgNPs and bentonite clay may be a suitable source of antimicrobial agents and should be investigated further for clinical applications. Importantly, the green produced AgNPs used in this study are biocompatible with the species studied, implying that they pose little environmental risk and toxicity. More research on the isolation of active chemicals from this plant species is being conducted.

## Figures and Tables

**Table 1:** Some of the physiochemical properties of bentonite.

Model substrate	Source	Primary particle size (nm)	PZC at pH	BET surface area (m^2^g^-1)^	Charge on particle	Particle size distribution
Bentonite	Sigma-Aldrich	114.56	7.5	850	Negative	Fine

## Data Availability

No data is linked with this publication.

## Additional Points

*Data Transparency.* All authors ensure that all data and materials as well as software application comply with field standards.
